# Acoustic Radiation Force Impulse Imaging in Benign and Malignant Breast Lesions

**DOI:** 10.7759/cureus.1301

**Published:** 2017-06-01

**Authors:** Jayapriya Jayaraman, Venkatraman Indiran, Kanakaraj Kannan, Prabakaran Maduraimuthu

**Affiliations:** 1 Radiodiagnosis, Sree Balaji Medical College and Hospital; 2 Sree Balaji Medical College and Hospital

**Keywords:** elasticity imaging techniques, breast, neoplasm, acoustic impulse, shear wave velocity

## Abstract

**Background:**

Elastography is a promising way to assess tissue differences regarding stiffness or elasticity, which has been historically assessed manually by palpation. Combined with conventional imaging modalities, shear wave elastography can potentially evaluate the stiffness of a breast lesion and consequently help detect malignant breast tumor from benign ones. The aim of this study was to evaluate the diagnostic role of shear wave elastography in breast lesions in the Indian population.

**Material and methods:**

Fifty patients presenting with breast lesions were included in the study. All the patients were subjected to B-mode ultrasound and elastography using shear wave with Virtual Touch Imaging (VTI^TM^) (Siemens Medical Solutions USA, Inc., PA, USA) and Virtual Touch Quantification (VTQ^TM^) (Siemens Medical Solutions USA, Inc., PA, USA) and the obtained data was analyzed using an appropriate statistical test (independent samples t-test).

**Results:**

In our study group of 50 patients, 34 were benign and 16 were malignant. VTI^TM^ showed a sensitivity of 97% and a specificity of 93% with a positive predictive value (PPV) of 97% for benign lesions. VTI^TM ^showed a sensitivity of 87.5 % and a specificity of 100% with a PPV of 100% for malignant lesions. VTQ^TM^ showed a sensitivity of 71.4% and a specificity of 100% with a PPV of 100% for benign lesions. VTQ^TM ^showed a sensitivity of 100% and a specificity of 100% with a PPV of 76.6% for malignant lesions.

**Conclusions:**

VTI^TM^ was more reliable as a diagnostic tool compared to VTQ^TM^ in benign lesions and both are equally reliable in identifying malignant lesions. Acoustic radiation force impulse (ARFI) plays a significant role as an adjuvant diagnostic tool to B-mode imaging for assessing breast lesions.

## Introduction

Breast lumps are a cause of great concern, irrespective of the age group. Breast cancer is the most important cause of cancer-related mortality among females in economically developing countries [1]. High frequency, high resolution ultrasound (US) helps in the evaluation of breast lumps, especially in younger females and those with dense breasts and apparently normal mammography. The most important function of a breast US is differentiating a cyst from a solid lesion. The high incidence of breast cancer and its slow evolution before diagnosis have led to research on new diagnostic techniques [1-3].

Elastography is a promising tool to assess tissue differences regarding stiffness or elasticity, which has been historically assessed manually by palpation. Elastography is very useful and precise in the evaluation of lesions situated in various organs including the breast [4-12]. Breast elastography provides supplementary information for characterization of lesions over conventional sonography and mammography. This technique, which provides information on the strain or hardness of a lesion, similar to a clinical palpation examination, has become a routine tool in addition to the diagnostic ultrasound during the last five years [3]. Two techniques now available for clinical use are compression-based strain elastography and shear wave elastography (SWE) [2].

The use of quantitative elastography with SWE improves the diagnostic accuracy in equivocal cases. SWE differentiates between benign and malignant lesions on the basis of their elasticity: benign lesions have elasticity similar to the surrounding tissue, while malignant lesions are harder than the adjacent tissue [13]. SWE is usually independent on the pressure exerted by the sonographer and provides better quantitative information and can be helpful for the characterization of breast lesions. Malignant tumors have reduced elasticity and also display larger dimensions on elastography due to the accompanying desmoplastic reaction [14-15]. Benign lesions appear similar to the adjacent tissues and have a smaller diameter than on B-mode ultrasonography (USG) images [4,16].

The objectives of this study were to derive the sensitivity and specificity, positive and negative predictive values of acoustic radiation force impulse (ARFI) in identifying benign and malignant lesions.

## Materials and methods

A prospective study was conducted from October 2013 to July 2015 following ethical approval from the Sree Balaji Medical College and Hospital Institutional Ethics Committee. Sixty-six patients with breast lumps who presented to the radiodiagnosis department and had imaging findings corresponding to Breast Imaging Reporting And Data System (BI-RADS) categories 0, 2, 3, 4, and 5 were included in the study. Patients with prior biopsy proven benign or malignant lesions were excluded from the study. A written consent was obtained from all the participants. Of these 66 patients, 16 of them did not have biopsy and histopathological diagnosis (eight of them were not willing and the other eight patients did not come back to the the department). Only 50 patients had histopathological diagnosis and were included in the analysis. Informed consent was obtained from all the patients.

All the studies were interpreted by two radiologists with 10 and 20 years experience in sonography and four years of experience in using ARFI. The patients with focal lesions (either cystic or solid) were assessed with conventional B-mode USG followed by SWE / ARFI. The study was done using Siemens ACUSON S 2000 ultrasound system (Siemens Medical Solutions, Mountain view, CA, USA). Shear wave elastography was done to obtain Virtual Touch Imaging (VTI^TM^) (Siemens Medical Solutions USA, Inc., PA, USA) and Virtual Touch Quantification (VTQ^TM^) (Siemens Medical Solutions USA, Inc., PA, USA) values.

The scoring system suggested by Itoh, et al. [14] was used. Score 1 signified deformability of the entire lesion; score 2 represented deformability of most of the lesion with some small stiff areas; score 3 represented deformability of the peripheral portion of the lesion with stiff tissue in the centre. Score 4 represented that the entire lesion is stiff and score 5 denoted that the entire lesion and surrounding tissue are stiff. If a lesion is classified between 1 and 3 it is considered benign; if classified 4 or 5 it is considered to be malignant. To calculate the sensitivity and specificity of elastography, lesions with elasticity scores 1–3 were classified as benign, while those with scores of 4 or 5 were classified as malignant. Hard lesions with VTQ above 9 m/s were depcited as x.xx m/s. Statistical analyses were performed using statistical software SPSS 16.0, SPSS Inc., Chicago, USA. The results were analyzed using the independent samples t-test. Sensitivity, specificity, positive and negative predictive values were obtained for the VTI and VTQ values.

## Results

**Figure 1:**
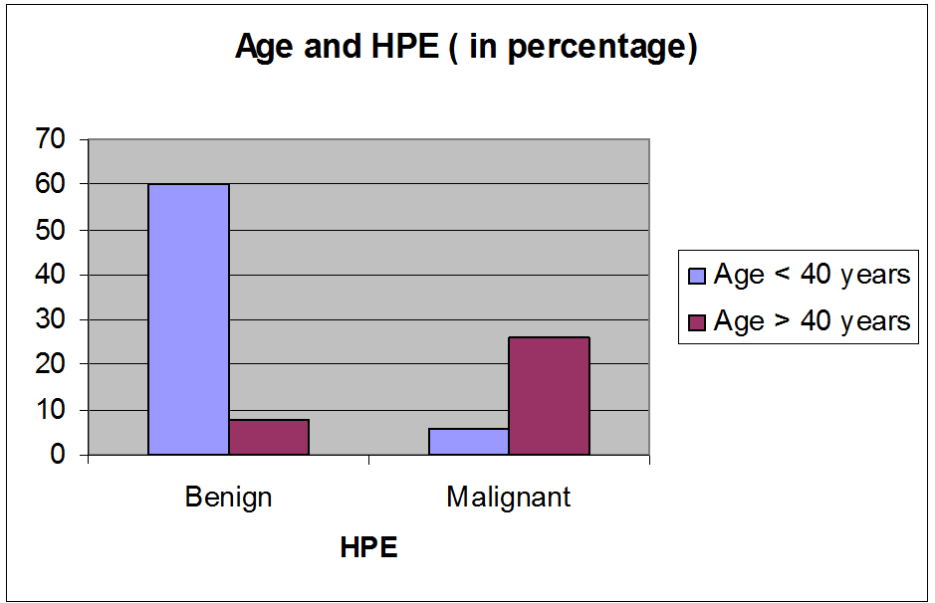
Age distribution and histopathology of the lesions Bar chart showing the age distribution and histopathology examination (HPE) of the lesions

**Figure 2:**
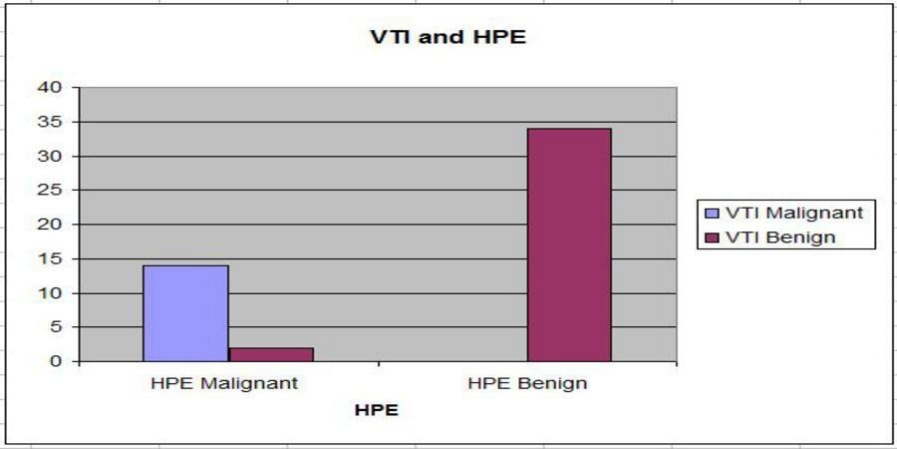
Virtual Touch Imaging diagnosis and histopathology of the lesions Bar chart showing the Virtual Tissue Imaging (VTI) diagnosis and histopathology examination (HPE) of the lesions

The age group of the 50 patients included in our study ranged from 15 to 71 years with a mean age of 40.56 (+\- 13.95) years. Thirty-three patients were less than or equal to 40 years of age and 17 patients were over 40 years of age. In our study group of 50 patients, 34 had benign and 16 had malignant lesions. In the age group of less than or equal to 40 years of age, 30 had benign lesions and three had malignant lesions. In the age group of more than 40 years of age, four had benign lesions and 13 had malignant lesions (Figure 1). The size of the lesions ranged from 5 mm to 54 mm. VTI correctly identified 14 of the 16 malignant lesions (a sensitivity of 87.5%) and all the 34 benign lesions (a specificity of 100%) (Figure 2). Our study showed a high positive predictive value (PPV) (100%) and a high negative predictive value (NPV) (94.4%) for VTI in identifying malignant lesions.

VTQ accurately identified all the 16 malignant lesions (a sensitivity of 100%) and 26 of the 34 benign lesions (a specificity of 76.4%) (Table 1). Our study showed a positive predictive value of 66.6% and a high negative predictive value of 100% for VTQ in identifying malignant lesions (Table 2). The study shows significant difference between the two parameters of ARFI imaging (VTI and VTQ) in differentiating benign and malignant lesions of the breast (P value < 0.001). VTI is more reliable as a diagnostic tool compared to VTQ in benign lesions, and both are equally reliable in identifying malignant lesions. Hence ARFI plays a significant role as an adjuvant diagnostic tool to B-mode imaging for assessing breast lesions. Hence routine ultrasound with ARFI correlation enhances the imaging diagnostic accuracy in categorizing benign and malignant breast lesions.

**Table 1:** Virtual Touch Quantification diagnosis and histopathology of the lesions Virtual Touch Quantification - VTQ, histopathology examination - HPE

	HPE Malignant	HPE Benign	
VTQ Malignant	16	8	24
VTQ Benign	0	26	26
	16	34	50

**Table 2:** Diagnostic accuracy of Virtual Touch Imaging and Virtual Touch Quantification Virtual Touch Imaging - VTI, Virtual Touch Quantification - VTQ

	VTI	VTQ
Sensitivity	87.5	100
Specificity	100	76.41
Positive predictive value	100	66.7
Negative predictive value	94.4	100

## Discussion

Breast cancer is one of the most frequently diagnosed cancers globally and also the main cause of cancer-related death among women. ARFI, as a new ultrasound-based elastography, provides quantitative and semi-quantitative measurements without invasiveness or radiation. Our results indicated that ARFI elastography has a high sensitivity and specificity for the diagnosis of malignant and benign breast lesions. The average shear wave velocity (SWV) of the 34 benign lesions (2.08 m/s) in this study was significantly lower than that of the 16 malignant lesions (6.28 m/s).

Different elasticity modes helped us to distinguish between benign and malignant solid breast lesions as stated in previous studies [12, 14, 17]. Solid malignant lesions were harder and stiffer than solid benign lesions as depicted in the articles earlier [6, 18-21]. The extra information derived from the assessment of elasticity is quite useful for atypical benign or malignant lesions (BI-RADS 3 or 4a) as outlined by the previous studies [12, 22-23]. Some studies show that the elastography could avert biopsy in case of BI-RADS 3-4a benign lesions in women with a high risk of breast cancer [16].

**Figure 3:**
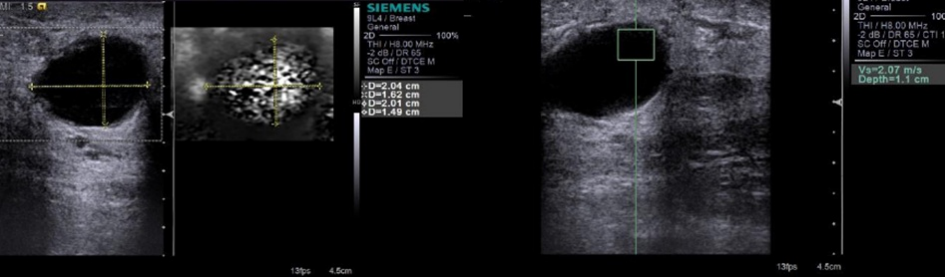
Cystic benign lesion on elastography B-mode shows a clearly cystic benign lesion with a VTI score of 1 and a VTQ of 2.07m/sec

**Figure 4:**
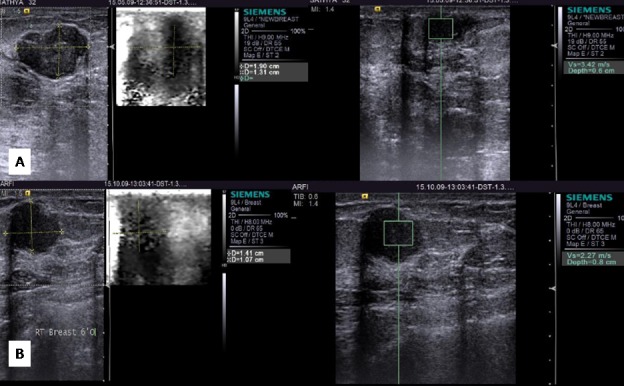
Fibroadenomas (A) B-mode shows a hypoechoic fibroadenoma with a VTI score of 2 and a VTQ of 3.42 m/sec. (B) B-mode shows another hypoechoic fibroadenoma with a VTI score of 3 and a VTQ of 2.27m/sec

**Figure 5:**
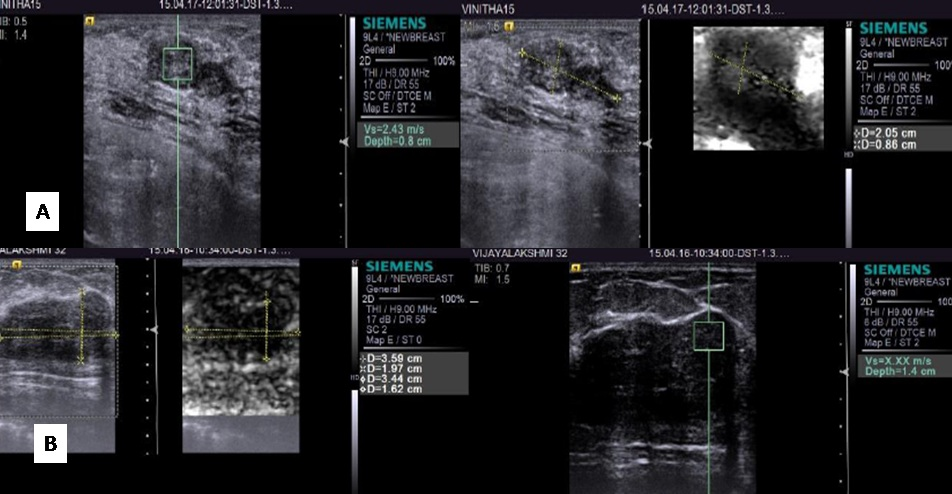
Fibroadenomas on elastography (A) B-mode shows a hypoechoic fibroadenoma with a VTI score of 2 and a VTQ of 2.43 m/sec. (B) B-mode shows another hypoechoic fibroadenoma with a VTI score of 3 and a VTQ of x.xx m/sec

**Figure 6:**
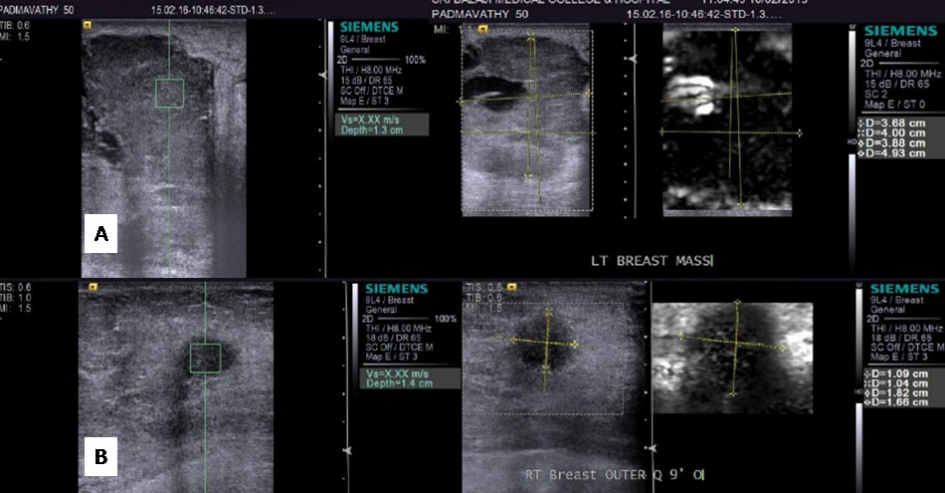
Malignant lesions on elastography (A) B-mode shows a solid lesion with cystic change with a VTI score of 5 and a VTQ of x.xx m/sec. (B) B-mode shows a small solid malignant lesion with a VTI score of 2 and a VTQ of x.xx m/sec

Elastography combined with B-mode imaging improved the specificity and sensitivity for differentiating benign and malignant lesions. Breast elastography is very useful to distinguish benign cysts with internal echoes from homogeneous solid lesions such like fibroadenoma. Cystic features are unequivocal on elastography with no internal register (Figure 3). Fine needle aspiration (FNA) could be avoided in cases with benign features on B-Mode imaging and typical cystic features on elastography. Lesions associated with benign micro calcifications showed significantly lower elasticity score than those with malignant micro calcifications. Hence, the VTI was more reliable with scores 3 denoting the benign nature whereas VTQ showed higher stiffness evidenced by increase in SWV as x.xx m/s (Figures 4-5). All malignant lesions in this study were more of solid nature and labeled as BI-RADS 4c and 5. The ARFI values were corresponding to scores 4 to 5 in VTI and higher unmeasurable SWV depicted as x.xx m/s (Figure 6). Two lesions that appeared benign on B-mode with smooth borders and mild vertical orientation gave higher tissue stiffness scores in ARFI and on correlation with histopathological examination (HPE) was proven to be malignant, and hence, ARFI has significantly played its part in upgrading the BI-RADS grading. Both VTQ and VTI increased the diagnostic confidence of a malignant lesion. Nevertheless, false negatives were also there. Soft lesions such as mucinous carcinoma, cystic carcinoma or inflammatory cancer contributed to the false negative lesions [24]. False positive cases were seen in fibrous lesions like long-standing fibroadenoma (Figure 5). Combining B-mode and ARFI yielded increased sensitivity than B-mode sonography alone.

Dan-Dan Li, et al. [1] analyzed the utility of ARFI elastography in differentiating malignant and benign breast lesions and derived cut-off values for benign and malignant lesions. The VTQ ranged widely from 2.89 to 6.71 m/s for benign lesions, while the VTI ranged from 1.37 to 1.66. Itoh, et al. [14] examined 111 nodules using real time elastography and found the mean score of elasticity to be significantly higher in malignant lesions (score 4.2 ± 0.9) than in benign lesions (score 2.1 ± 1). Giuseppetti, et al. [17] evaluated the potential usefulness of real time elastography in the study of 91 nodules using the Ueno score system [18] and established sensitivity and specificity of 79% and 89%, respectively. They remarked that the degree of elasticity was influenced by the size of the lesions as well as their histology. In their study to assess the role of elastography using ARFI in the diagnosis of solid breast lesions, Tozaki, et al. [19] found that the negative predictive value was 100%, as seen for VTQ in our study (Table 3). In another study, Tozaki, et al. [20] deduced that the mean shear wave velocity was significantly higher in malignant lesions than in benign lesions (4.49 m/s vs. 2.68 m/s). It was not possible to measure shear wave velocity in 23.5% of the malignant lesions, as seen in our study also. Meng, et al. [21] found that benign lesions showed lower mean area ratio and mean shear wave velocity (SWV) than in malignant lesions (mean area ratio of 1.08 ± 0.21 m/s vs. 1.99 ± 0.63 m/s and SWV of 3.25 ± 2.03 m/s vs. 8.22 ± 1.27 m/s, respectively) on ARFI. Bai, et al. [22] also found a significant difference in SWV between benign (2.25 ± 0.59 m/s) and malignant (5.96 ± 2.96 m/s) lesions with a sensitivity and specificity of 75.6% and 95.1%, respectively. Shear wave velocity measurement was not attainable in 63.4% of the malignant lesions.

Tozaki, et al. [23] found that visual elastographic images and measurement of Young's modulus had a sensitivity and specificity of 91.3% and 80.6% in distinguishing benign from malignant lesions. They concluded that simultaneously using both methods might help in better characterization of solid breast lesions. Athanasiou, et al. [24] compared the quantitative values of tissue elasticity with histologic findings and concluded that quantitative evaluation provided better information and characterization of breast lesions. Evans, et al. [25] compared ultrasound and real time shear velocity imaging in 53 solid nodules and found that real time shear velocity imaging yielded a higher diagnostic accuracy (a sensitivity of 97%, a specificity of 83%, a positive predictive value of 88 and a negative predictive value of 95%).

**Table 3:** Table showing the elastography values and diagnostic accuracy of elastography in various studies

Author	Qualitative /Quantitative imaging (VTQ)	VTI score	VTQ values	Diagnostic accuracy
Dan-Dan Li, et al. [1]	Qualitative and VTQ	1.37 to 1.66 ( benign lesions)	2.89 to 6.71 m/s ( benign lesions)	
Itoh, et al. [14]	Qualitative	4.2 ± 0.9 (in malignant lesions) 2.1 ± 1 (in benign lesions)		
Giuseppetti, et al. [17]	Qualitative			79% (Sensitivity) 89% (Specificity)
Tozaki, et al. [19]	VTQ		4.49 m/s (malignant lesions) 2.68 m/s (benign lesions)	100% (negative predictive value for malignant lesions)
Meng, et al [21]	VTQ		8.22 ± 1.27 m/s (malignant lesions) 3.25 ± 2.03 m/s (benign lesions)	
Bai et al. [22]	VTQ		2.25 ± 0.59 m/s (benign lesions) 5.96 ± 2.96 m/s (malignant lesions)	Sensitivity (75.6%) and Specificity of (95.1%)
Tozaki, et al. [19]	Qualitative (VTI)			Sensitivity (91.3%) and specificity (80.6%)
Evans, et al. [25]			28 kPa (range 18 to 44 kPa) – benign lesions; 140 kPa (range 29 to 293 kPa) – malignant lesions.	Sensitivity (97%) Specificity (83%), Positive predictive value (88 %) Negative predictive value of (95%)
Our study	VTI VTQ		2.08 m/s (benign lesions); 6.28 m/s (malignant lesions )	Specificity and Positive predictive value (100% for VTI); Sensitivity and Negative predictive value (100% for VTQ)

Our study reiterates the superiority of ARFI in identifying benign and malignant lesions in the Indian population, but the relatively small sample size is a limitation of our study. The size of region of interest (ROI) for measuring VTQ is fixed; hence, very small lesions could not be assessed separately as the surrounding breast parenchyma was also included, altering the VTQ values. Other general technical limitations of ARFI are a lack of adequate knowledge about the role of the size of the lesion, ideal measurement point within the lesion, the role of the adjacent tissue and the distance of the measurement point from the skin. The variability of SWV when repeatedly measuring the same region is also not clearly understood. Physical compression also seems to affect the measurements and can result in bias if varying degrees of compression are applied. Moreover, VTQ is not technically accurate, as measurements above 9 m/s cannot be made [15]. That very large lesions could not be included entirely in VTI is also a minor technical limitation.

## Conclusions

ARFI that measures elasticity without the need for physical compression is an excellent replacement for strain imaging. VTI was more reliable than VTQ. ARFI VTQ characterizes solid malignant breast lesions exquisitely on the basis of increased SWV. Better characterization of solid breast nodules, categorized as BI-RADS 3 and BI-RADS 4a is possible, with potential to avert unnecessary biopsies. Better identification of echogenic cystic lesions is another exciting possibility with elastography. Reduced elasticity in subcentimeter nodules should prompt an immediate biopsy rather than follow-up as advised presently. False positives and false negatives can occur in fibrous lesions like long-standing fibroadenoma and soft lesions like mucinous / cystic / inflammatory carcinoma, respectively. Hence, ARFI VTQ may be used as a supplement to conventional B-mode ultrasound, rather than as a stand-alone test.
